# The brain as an insulin-sensitive metabolic organ

**DOI:** 10.1016/j.molmet.2021.101234

**Published:** 2021-04-15

**Authors:** Joshua L. Milstein, Heather A. Ferris

**Affiliations:** 1 Center for Brain Immunology and Glia, University of Virginia, Charlottesville, VA, USA; 2 Department of Neuroscience, University of Virginia, Charlottesville, VA, USA; 3 Division of Endocrinology and Metabolism, University of Virginia, Charlottesville, VA, USA

**Keywords:** Insulin, Intranasal insulin, Mitochondrial metabolism, Insulin resistance, Alzheimer's disease

## Abstract

**Background:**

The brain was once thought of as an insulin-insensitive organ. We now know that the insulin receptor is present throughout the brain and serves important functions in whole-body metabolism and brain function. Brain insulin signaling is involved not only in brain homeostatic processes but also neuropathological processes such as cognitive decline and Alzheimer's disease.

**Scope of review:**

In this review, we provide an overview of insulin signaling within the brain and the metabolic impact of brain insulin resistance and discuss Alzheimer's disease, one of the neurologic diseases most closely associated with brain insulin resistance.

**Major conclusions:**

While brain insulin signaling plays only a small role in central nervous system glucose regulation, it has a significant impact on the brain's metabolic health. Normal insulin signaling is important for mitochondrial functioning and normal food intake. Brain insulin resistance contributes to obesity and may also play an important role in neurodegeneration.

## Highlights

• Insulin is an important regulator of brain metabolism.
• Brain insulin signaling helps regulate whole body metabolism.
• Brain insulin signaling may play an important role in the pathogenesis of Alzheimer's disease.

## 1 Introduction

With the introduction of insulin in 1921, diabetes, then a near uniformly fatal disease, became manageable. But along with an initial increase in lifespan, many of complications of diabetes became apparent. Early research focused on the organs most associated with morbidity and mortality, including the kidney and heart, as well as the classically insulin-sensitive tissues responsible for much of glucose homeostasis such as liver and skeletal muscle. As this research has led to increasingly longer lifespan for those affected by diabetes, it has become apparent that no organ, including the brain, is spared by the disease. The huge advances in molecular biological and neuroscience tools over the past 30 years have opened the way for a greater understanding of how the brain, a non-classical insulin-sensitive tissue, is impacted by diabetes. Intriguingly, the potential roles of insulin in the pathogenesis and treatment of some neurological diseases, including depression, cognitive decline, and Alzheimer's disease (AD), have expanded the brain insulin signaling research field beyond the confines of diabetes.

While the brain comprises only 2% of the human body's overall mass, it utilizes an estimated 20% of the body's glucose. For the most part, this glucose utilization is not dependent on insulin-stimulated translocation of glucose transporter 4 (GLUT4), as seen in classical insulin-sensitive tissues such as adipose tissue and skeletal muscle. As such, the brain was labeled early on as an insulin-insensitive organ whose glucose utilization was mediated through insulin-independent mechanisms. Since that designation, a vast amount of research has shown that the brain is, in fact, insulin-sensitive, despite its ability to uptake glucose being, for the most part, insulin-independent. The first hints at the brain's insulin sensitivity came from studies showing widespread expression of the insulin receptor (IR) in the brain. Since then, we have learned that the brain regions that harbor high levels of IR expression are some of those most known for their roles in cognition and feeding behaviors. In addition, it is now apparent that insulin and its close relative, insulin-like growth factor 1 (IGF1), are both able to influence brain metabolism and cellular function. This review presents what is currently understood about insulin's role in the brain, beginning with its transport across the blood–brain-barrier (BBB) and its signaling effects on cellular function and metabolism. We also discuss insulin's role in modulating the activity of different brain circuits and the resulting behavioral and metabolic implications arising from this. We also address cognitive impairment and Alzheimer's disease, which are associated with insulin resistance, and the potential for combatting them with intranasal insulin and insulin sensitizers.

## 2 Insulin receptor expression and signaling in the brain

### 2.1 Brain insulin receptor expression

As noted, a critical first step in recognizing the brain as an insulin-sensitive organ was the identification of IR expression in a variety of brain regions. Although the insulin receptor is found ubiquitously throughout the brain, its expression is at higher levels in select regions such as the cerebellum, cortex, and hypothalamus [1]. The receptor itself is primarily found in the plasma membrane and is composed of dimers of α and β subunits. It is important to note that neurons and glial cells such as astrocytes express different isoforms of the α subunit of the insulin receptor. Neurons express the IR-A isoform, which excludes exon 11, whereas glia predominantly express IR-B, which includes exon 11 [[2], [3], [4]]. This differs from peripheral tissues where the majority of IRα expression is the IR-B isoform. Also in contrast to the brain, IRα isoform expression patterns in the periphery are largely tissue-dependent rather than cell-type-dependent [5]. Despite this, there has not been evidence to suggest differing affinities for insulin between central and peripheral IRs. In general, studies assessing the affinities for the IR-A and IR-B isoforms for insulin have concluded that the IR-A isoform has approximately a 1–2 fold higher than to the IR-B isoform (EC_50_ calculations range from 0.4 to 6 nM depending on the study and methods used) [[6], [7], [8], [9], [10], [11]].

**Figure 1:**
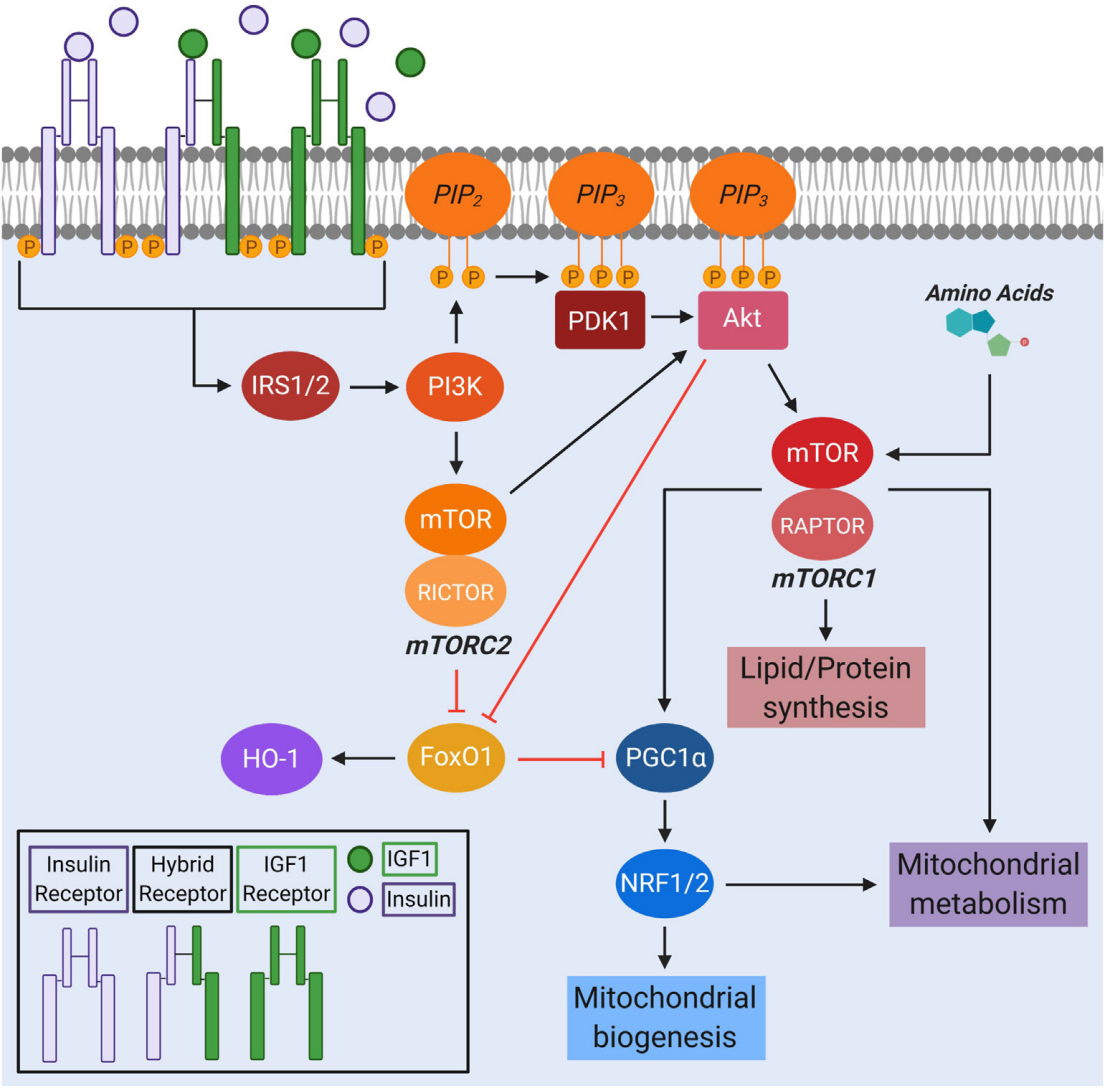
**Insulin signaling and mitochondria.** Binding of insulin and IGF1 to IR and IGF1R homo- and heterodimers (also known as hybrid receptors) initiates a signaling cascade that activates IRS1/2 and PI3K, which in turn activates mTORC2 and Akt. Both mTORC2 and Akt inhibit FoxO1 to prevent the transcription of HO-1. Activation of mTORC1 by Akt and amino acids promotes lipid and protein synthesis as well as mitochondrial metabolism and biogenesis through the PGC1α-NRF1/2 pathway. Akt: protein kinase B, FOXO1: forkhead box O1, HO: 1-heme oxygenase-1, IGF1: insulin-like growth factor 1, IRS1/2: insulin receptor substrate 1/2, mTORC1/2: mammalian target of rapamycin complex I/2, NRF1/2: nuclear respiratory factor 1/2, PGC1α: peroxisome-proliferator activated receptor coactivator-1α, PI3K: phosphatidylinositol 3-kinase.

To add even more complexity to receptor–ligand interactions, heterodimers consisting of an IR and a receptor for IGF1 (IGF1R) have been described in the brain and periphery [4,12] (Figure 1). As a result, there are five possible combinations of receptor dimerization between the IR and IGF1R, including the homodimers IR-A:IR-A, IR-B:IR-B, and IGF1R:IGF1R as well as the heterodimers IR-A:IGF1R and IR-B:IGF1R. These heterodimers, also referred to as hybrid receptors, seem to have increased affinity for IGF1 compared to insulin, although they are able to bind both of these hormones, along with IGF2, at varying affinities. IR-A:IGF1R and IR-B:IGF1R hybrid receptor EC_50_ values for insulin are widely variable between studies (∼1–350 nM for IR-A:IGF1R and ∼1–325 nM for IR-B:IGF1R), with some showing no difference in insulin binding and some showing a higher affinity by the IR-A:IGF1R hybrid receptor [8,11,[13], [14], [15], [16]]. Thus, more work is required to better clarify or confirm the different substrate-binding efficiencies of these two hybrid receptors. The extent to which IRs and IGF1Rs are in a heterodimer or homodimer conformation is believed to depend on the number of receptors in the tissue, as the energy required for producing either a hetero- or homodimer is equivalent [5,14,16]. How these heterodimers and homodimers differentially impact the brain's response to insulin requires additional research, although some work has been done to elucidate this in vitro. Specifically, Cai et al. [2] reported that the primary contributing factor to the downstream signaling effects of the IR and IGF1R is these two receptors' intracellular juxtamembrane domains, not the extracellular domain or substrate. While this study provides insight into what contributes to the differential signaling by IR and IGF1R homodimers after activation, the downstream effects of IR/IGF1R hybrid receptor signaling have not yet been well characterized.

### 2.2 Insulin production and transport across the BBB

Whether the brain produces its own insulin or if it is all or at least mostly pancreatic in origin has been controversial for decades. Pancreatic preproinsulin is processed in the endoplasmic reticulum and requires the expression and activity of multiple endopeptidases to cleave the C-peptide fragment from proinsulin. This generates the mature form of insulin that will subsequently be exocytosed in secretory granules by β cells in response to elevated blood glucose levels. Humans and rabbits have a single gene encoding for insulin, while rodents have two. Of the two *Ins* genes that lead to the production of preproinsulin in rodents, *Ins II* appears to be the one predominantly expressed by neurons [17,18]. In cultured rabbit neurons and glia, only neurons can secrete insulin into culture media [18]. While *Ins II* may be expressed in the brain in vivo, whether the endopeptidases that are required for secretion of the mature hormone product are expressed in the brain has been less well-demonstrated. Overall, data suggesting that the human brain produces and secretes significant amounts of insulin locally are lacking, and it is largely believed that the vast majority of insulin found in the brain parenchyma originates from the pancreas. To allow for this, insulin transport from the blood into the brain must occur via passage through the BBB and/or the blood-cerebrospinal fluid (CSF) barrier.

The insulin concentration in the CSF is significantly lower than that found in the blood. Multiple studies have shown that the CSF insulin concentration increases much slower and peaks at much lower levels than that of plasma insulin levels during hyperinsulinemic-euglycemic clamps [6,[19], [20], [21]]. Together with the fact that insulin is a 51-amino acid peptide, these findings suggest that insulin's transport into the brain is not through diffusion and instead requires active mechanisms. These mechanisms have not been fully elucidated, but studies using radiolabeled insulin have found that insulin quickly localizes to the brain endothelial cells, which can uptake insulin in an IR-dependent manner [6,[22], [23], [24]]. Confirming a role of endothelial IR in insulin transport across the BBB, endothelial cell-specific IR knockout (IRKO) mice intravenously injected with insulin have reduced downstream insulin signaling in the hippocampus, hypothalamus, and frontal cortex [25]. Insulin can also be transported across circumventricular organs such as the median eminence, where the BBB vessels are fenestrated. Here, insulin may be brought into the CSF or interact with specialized ependymal cells called tanycytes that facilitate receptor-mediated endocytosis of insulin for transport to neurons in the hypothalamus [6,26].

Notably, there is controversy regarding measuring CSF insulin concentrations to extrapolate brain parenchymal insulin levels. CSF insulin has been used as a proxy for tissue insulin given the relative ease of collection; however, insulin levels in the CSF are very low and may not reach levels needed to induce insulin signaling in the brain [6,19,20]. Complications with measuring brain insulin levels directly via microdialysis make interpreting these data difficult. An in-depth discussion of insulin transport and confounding insulin concentration measurements between compartments can be found in a recent review by Gray and Barrett [6].

### 2.3 Insulin signaling and glucose uptake in the brain

Once it has entered the brain, insulin binds to its receptor and initiates a series of phosphorylation events. First, an autophosphorylation event occurs on the intracellular tail of the IR, recruiting the insulin receptor substrates 1 and 2 (IRS1 and IRS2). Tyrosine phosphorylation of IRS1/2 leads to downstream activation of the kinases phosphatidylinositol 3-kinase (PI3K), protein kinase B (Akt), and mammalian target of rapamycin (mTOR) (Figure 1) [27,28]. This PI3K/Akt/mTOR signaling pathway impacts a wide variety of cellular functions, including synaptic plasticity, cholesterol synthesis, neuronal survival, and neurotransmitter trafficking [[29], [30], [31], [32]]. Insulin is also able to modulate cell growth and proliferation through induction of Shc and its downstream targets Ras, ERK, and mitogen-activated protein kinase (MAPK). Although insulin can promote this pathway, it appears to be more active in response to IGF1R signaling compared to IR signaling [2]. In addition, some evidence suggests that the different IRα isoforms promote different downstream signaling, with the IR-A isoform preferentially activating the mitogenic Shc/Ras/ERK/MAPK pathway and the IR-B isoform activating the PI3K/Akt/mTOR pathway [4,16].

The primary difference between peripheral and brain insulin signaling is the regulation of glucose transporters. In peripheral tissues, insulin-mediated Akt activation induces translocation of glucose transporter 4 (GLUT4) from vesicles to the plasma membrane to facilitate glucose uptake from the blood [33]. However, in the brain, the expression of GLUT4 is limited to specific brain regions such as the hippocampus and hypothalamus, resulting in a much smaller impact on glucose uptake [34,35]. Thus, it is primarily the glucose uptake effects of insulin signaling, rather than the signaling pathways themselves, which differ between the brain and periphery.

In the brain, glucose import from the circulation is primarily mediated through the insulin-insensitive GLUT1, which is expressed by endothelial cells and astrocytes at the BBB [[36], [37], [38]]. Within the brain parenchyma, GLUT3 and GLUT1, both of which are considered insulin-insensitive, are expressed widely by neurons and glial cells, respectively. There is evidence to support some function of insulin in regulating brain glucose uptake. Astrocyte-specific IRKO in adult mice results in decreased CSF glucose levels after peripheral glucose injection [39]. Furthermore, in vivo ^18^FDG-PET imaging of astrocyte-specific IRKO mice shows decreased glucose uptake in the brain and this effect coincides with diminished GLUT-1 mRNA expression [39]. There is also evidence that insulin can indirectly induce GLUT3 translocation to the plasma membrane in neurons to allow glucose uptake [40], suggesting that insulin plays a role in modulating glucose transporter expression in the brain, despite the limited expression of insulin-sensitive GLUTs. Fernandez et al. [41] identified a putative mechanism by which astrocytes can uptake glucose through GLUT1 translocation in response to concurrent signals from insulin and IGF-1. Together, these data indicate that insulin signaling may play an important role in regulating glucose uptake into the brain through non-traditional pathways.

Despite these findings, most glucose uptake by the brain is not regulated by insulin signaling. While this holds true for brain glucose metabolism as we currently understand it, insulin plays a critical role in intracellularly modulating metabolic activity through its regulation of metabolic signaling pathways.

### 2.4 Control of cellular metabolism by insulin

Diseases related to impaired insulin signaling, commonly referred to as insulin resistance, are predominantly associated with altered metabolic function. As such, it is not surprising that insulin affects many aspects of cellular and mitochondrial metabolism, not only in the periphery, but also in the brain. As the primary producers of cellular ATP, mitochondria are critical in maintaining metabolic homeostasis, and mitochondrial dysfunction is observed in many metabolic diseases that are characterized by insulin resistance.

As mentioned, the insulin signaling cascade is associated with the activation of Akt, which leads to assembly of mTORC1. mTORC1 signaling is critical for regulating protein, lipid, and fatty acid synthesis as well as mitochondrial metabolism [[42], [43], [44], [45]]. In terms of its metabolic impact, mTORC1 is integral to mitochondrial oxidative metabolism and biogenesis through its control of peroxisome-proliferator activated receptor coactivator (PGC)-1α and the transcription factors nuclear respiratory factors 1 and 2 (NRF1/2) (Figure 1) [46,47]. In addition to activation by insulin through Akt, mTORC1 can be activated by increased levels of amino acids [[48], [49], [50]]. When mTORC1 is activated by either insulin or amino acids, production of nuclear-encoded mitochondrial proteins is stimulated. These are then incorporated into a variety of mitochondrial metabolic pathways, including the tricarboxylic acid (TCA) cycle, fatty acid β oxidation (FAO), and the electron transport chain complexes [51]. Further, mTORC1 drives a metabolic shift from oxidative phosphorylation (OXPHOS) to glycolysis during cell growth and development. The ability of mTORC1 to sense amino acids and regulate both mitochondrial metabolism and biogenesis places it as a central player controlling cellular metabolism and nutrient sensing. This may be important for regulating metabolism in different brain cell types, both under normal conditions and metabolic stress. For example, mTOR-driven autophagy in response to metabolic stress appears to be more robust in astrocytes than neurons [52]. Therefore, it is possible that the regulation of metabolism by mTORC1 signaling may differ between these cell types. Because astrocytes utilize glycolysis and FAO as their primary means of energy production while neurons preferentially use OXPHOS [[53], [54], [55], [56]], differences in mTOR signaling may play a role in establishing these metabolic phenotypes. However, this comparison and whether the variable mTOR signaling in these cells differentially affects their mitochondrial response to nutrient deprivation have yet to be addressed.

mTOR is also found in a second complex, mTORC2, which acts through Akt to promote cell proliferation and survival. mTORC2-mediated Akt activation also negatively regulates forkhead box O1 (FoxO1) (Figure 1). FoxO1 promotes the transcription of heme oxygenase-1 (HO-1). Excess hepatic HO-1 expression in the liver impairs mitochondrial OXPHOS and FAO [57] by decreasing mitochondrial biogenesis [57,58]. Therefore, insulin's activation of Akt inhibits FoxO1-dependent HO-1 transcription and prevents HO-1 hyperactivation-induced mitochondrial dysfunction in the liver. Whether this same process occurs in the brain has not been determined. However, there is evidence suggesting an important role of insulin-mediated regulation of FoxO1 in controlling food intake, insulin sensitivity, and glucose homeostasis. Mice with constitutively active nuclear FoxO1 have elevated food intake and associated obesity, while mice with deletion of FoxO1 in hypothalamic neurons have diminished food intake [[59], [60], [61], [62], [63], [64]].

Insulin's other major signaling pathway, which involves activation of Ras/ERK/MAPK, also modulates mitochondrial homeostasis and function. In in vitro experiments using a hypothalamic neuronal cell line, Wardelmann et al. [65] found that insulin acts through ERK to induce the expression of the mitochondrial chaperones heat shock protein (Hsp) 60 and Hsp10. They further identified this pathway as a potential mediator of insulin-induced mitochondrial respiration, as inhibition of ERK signaling alleviated the induction of mitochondrial respiration due to insulin treatment. Of note, they found the same response with IGF1 acting through the IGF1R. Whether these effects in vivo are primarily driven by IGF1R signaling or IR signaling remains to be determined. Altogether, brain insulin action has clear roles in regulating cellular metabolism despite its limited impact on modulating glucose uptake into the brain.

## 3 Brain circuitry and behavior in response to insulin

### 3.1 Insulin signaling in neuronal populations

As previously discussed, one of the prominent effects that insulin exerts on the brain is regulating feeding behaviors. This is done, in part, through its binding to IRs on pro-opiomelanocortin (POMC) and agouti-related protein (AgRP) neurons in the arcuate nucleus of the hypothalamus. AgRP neurons are orexigenic and promote hunger while POMC neurons are anorexigenic and promote satiety. Thus, the individual activities of these neuronal populations oppose each other to modulate hunger and food intake. On balance, intracerebroventricular infusion of insulin reduces food intake in both fasted and non-fasted rodents and results in weight loss [6,[66], [67], [68], [69]]. However, insulin's role in these subpopulations of neurons extends beyond food intake and into the periphery through regulation of peripheral metabolism.

For example, central insulin resistance alters glucose sensing in hypothalamic neurons, leading to an impaired sympathetic outflow in response to hypoglycemia [70,71]. Insulin control of sympathetic outflow also seems to participate in body temperature regulation. When IR is knocked out from the brain, mice become hypothermic [72]. In contrast, intranasal delivery of insulin in humans promotes thermogenesis [73]. At least in rodents, this effect is mediated by sympathetic activation of brown fat [74].

**Figure 2:**
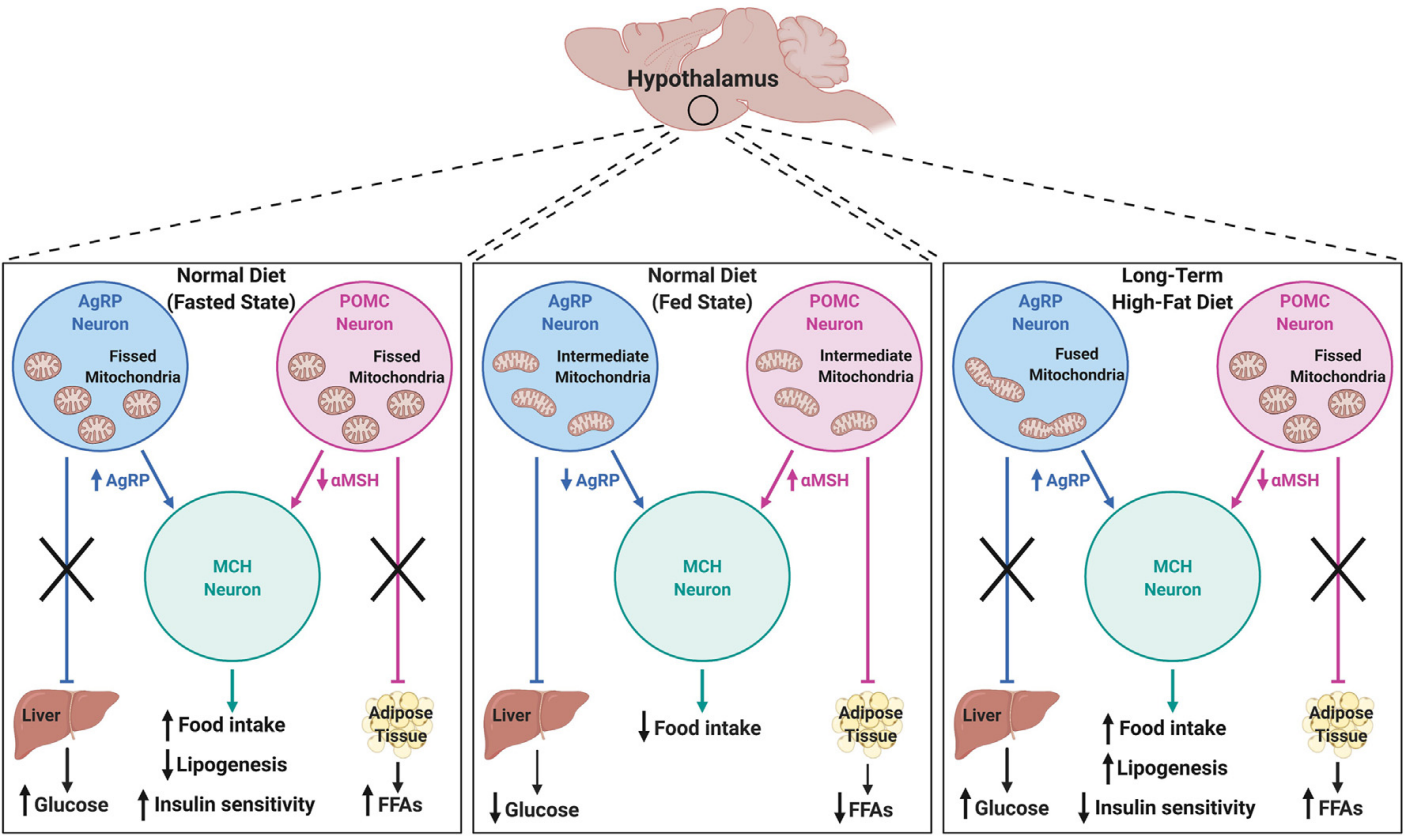
**Hypothalamic regulation of whole-body metabolism under different feeding states.** Fasting induces mitochondrial fission in both AgRP and POMC neurons in the arcuate nucleus of the hypothalamus. AgRP neurons produce more AgRP protein, while POMC-derived αMSH decreases. These combined effects decrease the activity of MCH neurons in the paraventricular nucleus of the hypothalamus to increase food intake. AgRP and POMC regulation of hepatic glucose production and adipose tissue lipolysis is diminished to allow for elevated circulating levels of blood glucose and FFAs, resulting in reduced fat mass and higher insulin sensitivity. Under fed conditions, mitochondria in AgRP and POMC neurons are in an intermediate state, with a balance between mitochondrial fusion and fission. AgRP release is diminished and αMSH is enhanced, promoting MCH neuronal activity and satiety. IR signaling in AgRP and POMC neurons inhibits hepatic glucose production and lipolysis, respectively. In response to long-term HFD feeding, AgRP neuronal mitochondria enter a fused state, whereas mitochondria in POMC neurons are fissed. AgRP neurons upregulate the production of AgRP protein, while αMSH production by POMC neurons is impaired, inhibiting MCH neurons and increasing food intake. Impaired IR action on AgRP and POMC neurons leads to deficits in these neurons' ability to inhibit hepatic glucose production and lipolysis, resulting in elevated fat mass and diminished insulin sensitivity. AgRP: agouti-related protein, FFAs: free fatty acids, MCH: melanin-concentrating hormone, αMSH: α-melanocyte-stimulating hormone, POMC: pro-opiomelanocortin.

Further evidence delineating the impact that insulin signaling in hypothalamic neurons has on peripheral metabolism comes from cell-specific IRKO experiments in AgRP and POMC neurons. These studies demonstrated that insulin signaling in AgRP neurons regulates hepatic glucose production, while insulin signaling in POMC neurons affects adipose tissue lipolysis (Figure 2) [75,76]. Supporting this, insulin injection directly into the CNS increases insulin sensitivity in the liver while also stimulating lipogenesis and fat accumulation [77,78]. However, insulin's role in POMC neuronal control of hepatic glucose production has been controversial due to dichotomous results showing that insulin can either inhibit POMC neuron activity [76,[79], [80], [81]] or promote it [[82], [83], [84]]. Although more work is needed to better understand these findings, a series of recent studies from the Tiganis group [[85], [86], [87]] shed some light on this issue by identifying T cell protein tyrosine phosphatase (TCPTP), whose expression increases during fasting and decreases post-prandially. This phosphatase appears to be able to determine whether insulin is inhibitory or excitatory to POMC neuronal firing and control of hepatic glucose production. Others have shown through single-cell profiling that POMC and AgRP neuronal populations in the hypothalamus are heterogeneous and these subsets may act differently in response to nutrient availability [[88], [89], [90]], further suggesting that the regulation of these neuronal populations and their effects in the brain and periphery are much more complex than currently understood.

This complexity is likely reflected in human clinical trials of intranasal insulin. This delivery method, which allows for direct delivery of insulin to the brain, thus avoiding the potential for hypoglycemia, results in suppressed food intake in most trial participants [91,92]. However, one study that treated patients with intranasal insulin for 8 weeks found that while men lost body fat with the treatment, women did not [93]. This may represent a sex-based difference in the balance of hypothalamic control by centrally acting insulin of food intake vs stimulation of peripheral lipogenesis. Altogether, these various studies demonstrate that in addition to insulin's important role in regulating brain metabolism, there are multiple peripheral homeostatic functions that are fine-tuned by insulin signaling in the brain.

Convincing evidence supporting a specific role of insulin signaling in the brain in cognitive or affective behaviors has been scarce until more recently. The commonly cited and used nestin-Cre IRKO (NIRKO) mouse shows some anxiety behaviors later in life [94,95]. This mouse is sometimes mistakenly referred to as a neuron-specific knockout, but nestin is an intermediate filament expressed during development by all neural progenitor cells. As such, the NIRKO mouse is a model of whole-brain IRKO (excluding microglia) rather than a neuron-specific deletion. Additional evidence implicates IR/IGF1R signaling in both the hippocampus and amygdala in behavior. Using adeno-associated virus (AAV)-Cre injection into both of these brain regions, Soto et al. [96] found that IR/IGF1R double-KO (DKO) in either brain region elevated anxiety behaviors and impaired systemic glucose homeostasis compared to mice injected with a control AAV. Further, hippocampal IR/IGF1R DKO mice had impaired spatial memory, whereas DKO in the amygdala dysregulated brown adipose tissue thermogenesis [96]. These findings were consistent with a previous report that showed injection into the hippocampus of a lentiviral construct expressing an IR antisense sequence to downregulate IR expression specifically in this region negatively affected long-term potentiation and spatial memory [97]. While these two methods may have been able to decrease IR expression in the hippocampus and amygdala, they were not able to distinguish cell-type-specific effects of IR or IR/IGF1R DKO in these brain regions. It will be critical to determine the individual contributions of insulin and IGF1 in long-term potentiation and spatial memory formation in the hippocampus and whether IR/IGF1R homodimers and heterodimers have distinct roles in these processes.

#### 3.1.2 Mitochondrial dynamics regulate hypothalamic neuron activity

Mitochondrial dynamics, among other aspects of mitochondrial biology, include fission and fusion of mitochondria and these processes are imperative for mitochondrial quality control and adaptation to the cell's redox and energetic state. As the names imply, mitochondrial fusion is the process in which two discrete mitochondria combine into a single mitochondrion or when a single mitochondrion integrates into a mitochondrial network. In contrast, mitochondrial fission describes the separation of a single mitochondrion into two discrete organelles or the removal of a mitochondrion from the mitochondrial network. Mitochondrial fusion is primarily mediated by the proteins mitofusin 1 (MFN1), MFN2, and Opa1, whereas fission is mediated by the proteins dynamin-related protein 1 (Drp1), mitochondrial fission factor (MFF), and Fis1. Generally, when the cell is under energetic stress or nutrient deprivation, mitochondria will fuse and become elongated in an attempt to maximize energy production [98,99]. However, this response to stress may not be sustainable, as the increase in OXPHOS from these mitochondria may also augment ROS production and eventual mitochondrial damage [100]. Mitochondrial fission aids mitochondrial quality control by ensuring that old or damaged mitochondria are sequestered and isolated off of the mitochondrial network to be degraded through selective autophagy, termed mitophagy. In this way, fission is able to respond to increases in ROS or decreases in ATP production to mitigate these and limit downstream damage. These basic aspects of mitochondrial biology and homeostasis have been identified as imperative for maintaining mitochondrial adaptations to nutrient availability and neuronal activity in both AgRP and POMC neurons [[101], [102], [103], [104]], which has direct implications for regulating whole-body insulin sensitivity and glucose homeostasis.

Under fasted conditions, mitochondria in both AgRP and POMC neuron populations undergo increased fission levels, which coincide with decreased POMC activity and increased AgRP activity [101,103,104]. After feeding, mitochondrial fusion is upregulated in these cells, resulting in an intermediate phenotype of the mitochondrial network that is associated with diminished AgRP activation and enhanced POMC activation [[101], [102], [103], [104]]. It is clear that the regulation of mitochondrial fission and fusion is required for proper activation of POMC neurons under fed conditions. This comes from mouse studies demonstrating that POMC-specific KO of the fission regulator Drp1 or either of the fusion regulators MFN1/2 leads to altered POMC activity and impaired food intake and whole-body glucose tolerance [[102], [103], [104]] (Figure 2). Mitochondrial fusion in POMC neurons may also be involved in controlling pancreatic glucose-stimulated insulin secretion by these cells, as POMC-specific MFN1 KO increased sympathetic outflow to the pancreas, resulting in reduced insulin secretion [103]. Taken together, mitochondrial dynamics in both AgRP and POMC neurons are not only affected by nutrient availability, but are also themselves important for these hypothalamic neuronal populations to function regularly and therefore modulate feeding behaviors, insulin sensitivity, glucose homeostasis, and fat storage throughout the body (Figure 2).

### 3.2 Astrocyte insulin signaling

Astrocytes are integral for BBB integrity and neuronal metabolic and redox homeostasis [105]. It has become increasingly apparent that insulin signaling in astrocytes is imperative for these processes and modulating behavior and whole-body glucose homeostasis [39,106]. Whereas NIRKO mice do not have any behavioral abnormalities until later in life, mice with astrocyte-specific IRKO exhibit depressive and anxiety behavioral phenotypes at an earlier age [106]. These effects have been attributed to impaired dopamine release and signaling in the nucleus accumbens [106]. Further, these mice are hyperphagic and have impaired peripheral glucose tolerance, insulin sensitivity, and POMC neuronal firing in response to glucose [39]. IRKO from astrocytes in the hypothalamus shows similar whole-body effects, suggesting that insulin signaling in hypothalamic astrocytes may serve a crucial role in modulating food intake and glucose homeostasis [39].

Interestingly, astrocyte IGF1 signaling also seems to be involved in learning and memory, as KO of IGF1R from astrocytes in mice impairs working memory [107]. Cultured astrocytes respond to both insulin and IGF1 by increasing GLUT1 expression and glucose uptake [41]. Individual roles of insulin and IGF1 signaling in modulating astrocyte metabolic activity have also been described in in vitro systems. IGF1R KO in primary astrocyte cultures reduces their basal oxygen consumption rate and adenylate energy charge [107], a measure of the cell's energetic state that accounts for cellular AMP, ADP, and ATP levels. Similar to cultured neurons, treating primary cortical astrocytes with insulin suppresses H_2_O_2_ production and increases mitochondrial ATP production, suggesting that insulin-stimulated mitochondrial respiration occurs in both of these cell types [108]. IRKO astrocytes in vitro have diminished glycolytic activity as evidenced by decreased glucose uptake and l-lactate release, which appears to be due to a switch in their metabolic phenotype from glycolysis to FAO [39]. Because astrocytes contribute to neuronal metabolic homeostasis and respond to neuronal firing by releasing lactate [56,105,109], these findings may have implications for astrocyte insulin signaling in maintaining neuronal metabolism and brain metabolic defects observed in insulin-resistant conditions. While this phenomenon also appears to be present in humans based on modeling nuclear magnetic resonance imaging data [110], whether this astrocyte metabolic phenotype switch is altered in vivo during insulin resistance or neurodegenerative disease has not been assessed. Altogether, these data add astrocyte insulin signaling to the ever-growing list of ways that astrocytes and neurons interact and impact each other's function.

## 4 Brain insulin resistance and metabolism in disease

### 4.1 Molecular contributors to brain insulin resistance

A number of mechanisms have been proposed as potential factors in the development of brain insulin resistance. When we consider the complexity and heterogeneity of diseases associated with insulin resistance, it becomes clear that there is undoubtedly an interplay between multiple mechanisms in vivo. For example, consuming a high-fat diet, living a sedentary lifestyle, and genetic predisposition are all risk factors for developing insulin resistance and type 2 diabetes (T2D) in humans. Although many of these are also risk factors for developing cognitive decline and AD, AD's brain insulin resistance is also present in individuals without diabetes [111].

We next explore some of the mechanisms thought to contribute to brain insulin resistance, mostly through the lens of a high-fat diet in mice. Mouse studies of HFD-induced obesity and insulin resistance have found that the composition of the HFD, including the source of fat, has a significant impact on the degree of insulin resistance. HFD can result in increases in free fatty acids (FFAs), ceramides, phosphatidic acid, and diacylglycerols, all of which have been implicated in peripheral insulin resistance [112]. Humans rarely if ever eat a diet that is as consistent and molecularly defined as what mice are fed in HFD studies. Despite these considerations, much can be learned from mouse studies about how insulin resistance develops, both throughout the body and in the brain, and how this contributes to metabolic defects and neurodegeneration. Additionally, while not discussed in more detail, it is important to note that there is likely at least some contribution of impaired insulin transport into the brain in response to a HFD and during neurodegenerative diseases associated with brain insulin resistance. The extent to which disrupted insulin transport into the brain or any of the individual molecular mechanisms described contribute to cognitive impairment and insulin resistance in the brain compared to any of the other mechanisms is unknown. It is critical to keep all these considerations in mind when discussing and studying brain insulin resistance in any given neurological context.

#### 4.1.1 Fatty acids and inflammation

**Figure 3:**
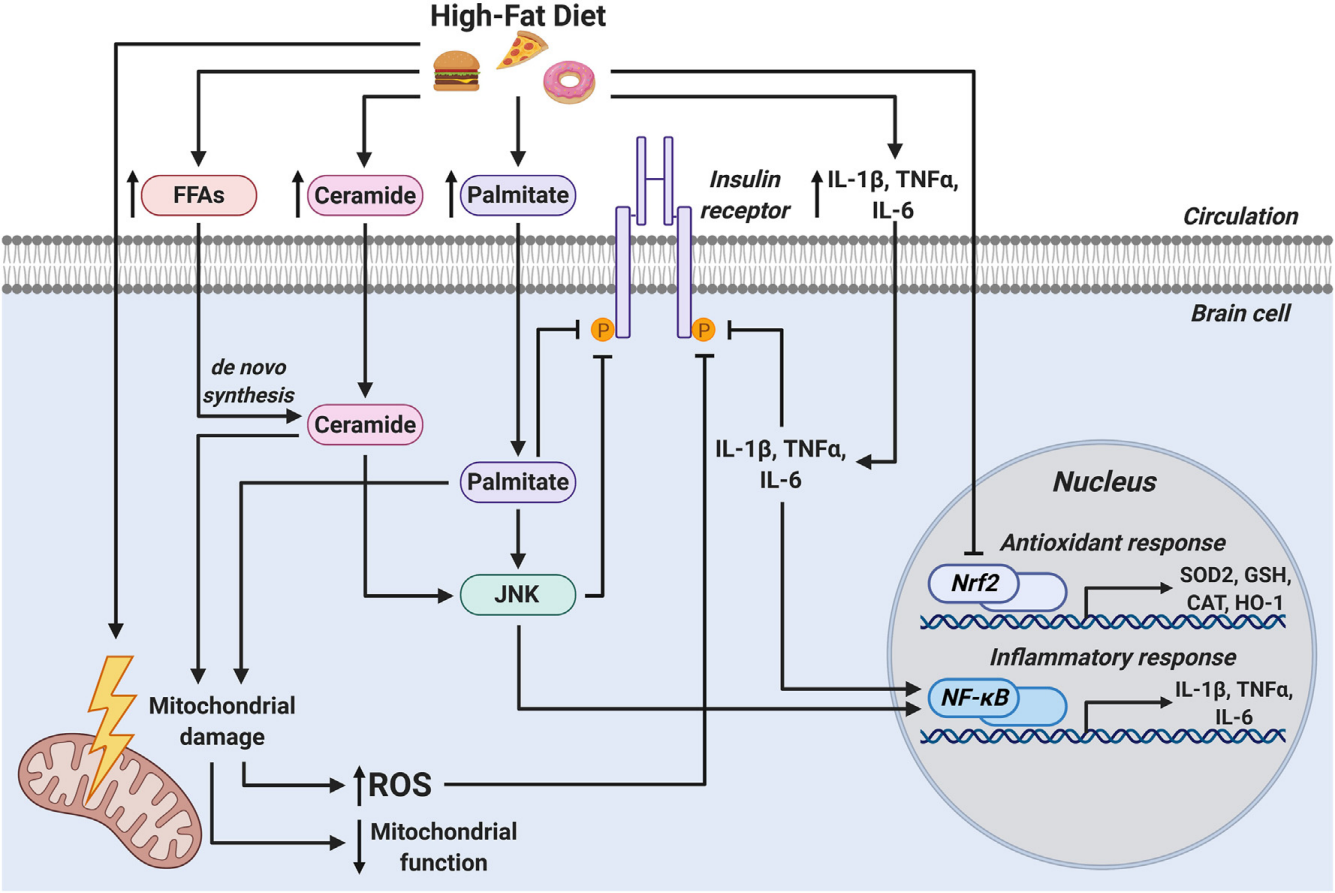
**Mechanisms of brain insulin resistance.** High-fat diet feeding leads to elevated circulating levels of FFAs, ceramides, palmitate, and inflammatory cytokines. All these are transported into the brain, where they impair insulin receptor signaling, activate ER stress signaling through JNK, and initiate an inflammatory response via NF-κB signaling. HFD feeding, palmitate, and ceramide induce mitochondrial damage, resulting in elevated ROS production and diminished mitochondrial function. The increase in ROS production coupled with HFD-induced impairment of the Nrf2-driven antioxidant response culminates in oxidative stress, which further exacerbates insulin resistance. CAT: catalase, GSH: glutathione, FFAs: free fatty acids, HFD: high-fat diet, HO: 1-heme oxygenase-1, IL-1β: interleukin-1β, IL-6: interleukin-6, JNK: c-Jun N-terminal kinase, NF-κB: nuclear factor kappa-light-chain-enhancer of activated B cells, Nrf2: nuclear factor erythroid 2-related factor 2, ROS: reactive oxygen species, SOD2: superoxide dismutase 2, TNFα: tumor necrosis factor α.

One of the primary mechanisms proposed for the development of brain insulin resistance is the accumulation of damaging FFAs and ceramides in the brain because of HFD intake. Indeed, chronic brain infusion of saturated fatty acids such as palmitic acid (PA) leads to insulin resistance [113]. Elevated levels of circulating FFAs induce the synthesis of ceramides in the brain [[114], [115], [116]]. Excessive brain ceramides induce inflammation by activating the nuclear factor kappa-light-chain-enhancer of activated B cell (NF-κB) pathway, which coordinates the transcription of proinflammatory cytokines such as interleukin-1β (IL-1β), tumor necrosis factor α (TNFα), and IL-6. These inflammatory mediators not only impair insulin signaling on their own, but also activate c-Jun N-terminal kinase (JNK), a signaling molecule involved in the endoplasmic reticulum (ER) stress response that disrupts brain insulin signaling (Figure 3) [115,117,118]. Importantly, Schell et al. [119] demonstrated that varied compositions of HFDs have differential effects on the induction of insulin resistance, JNK activation, and mitochondrial function in the hypothalamus. This research highlights the importance of matching the macronutrient composition of control and experimental diets and testing different types of HFDs (Western vs Mediterranean) to better elucidate which HFD components may influence the onset of insulin resistance.

Palmitic acid, which is the most abundant saturated fatty acid in most HFDs used for rodent research, is increased in the cerebrospinal fluid and brains of individuals with obesity [116,120,121] and the hypothalamus of mice fed a long-term HFD [118]. Treating neurons in vitro with PA leads to insulin signaling deficits, inflammation, and JNK activation in these cells [118,119]. Primary astrocytes treated with PA have similarly elevated astrogliosis and inflammatory cytokine expression [118]. Interestingly, inducible KO of a key inducer of NF-κB, IKKβ, from astrocytes after the onset of HFD-induced obesity and astrocyte reactivity reduces food intake and prevents further weight gain in these mice [122]. Furthermore, mice with IKKβ KO in their astrocytes have elevated energy expenditure, glucose tolerance, and insulin sensitivity, all of which coincide with diminished hypothalamic inflammation [122]. Supporting this, overexpression of IKKβ and NF- κB led to weight gain coupled with glucose intolerance and insulin resistance [123]. These studies suggested that insulin resistance, induced by either a HFD or elevated PA concentrations, is detrimental to astrocytes due to induction of an inflammatory state. Indeed, astrogliosis has also been observed in obese humans [24,[124], [125], [126]]. Whether the inflammatory response of astrocytes to a HFD plays a direct role in influencing whole-body energy expenditure, food intake, glucose tolerance, and insulin sensitivity or if it instead acts indirectly by influencing neuronal populations that are involved in controlling these processes remains unclear.

#### 4.1.2 Oxidative stress

As a natural byproduct of mitochondrial oxidative metabolism and some enzymatic reactions, ROS have been implicated in the control of several cellular functions, including cell death, cell signaling, induction of antioxidant responses, and regulation of mitochondrial metabolism. Indeed, mitochondrial ROS are critical for many major signaling pathways, including NF-κB, JNK, and insulin among others [[127], [128], [129], [130], [131]]. Excess free radicals are normally neutralized by a variety of antioxidants and enzymes that together form the antioxidant response. While more nuanced and extensive than discussed in this review, the antioxidant response is largely controlled by the transcription factor nuclear factor erythroid 2-related factor 2 (Nrf2), which regulates the expression and activity of many key antioxidants and antioxidant biosynthesis pathways [132] including superoxide dismutases (SODs), glutathione (GSH), HO-1, and catalase (CAT). The short polypeptide antioxidant GSH is used as a cofactor for antioxidant enzymes such as glutathione peroxidases that are critical for reducing ROS and lipid peroxides [133]. In addition, many of these are localized within mitochondria to regulate mitochondrially derived ROS. All these antioxidants maintain cellular redox balance and prevent oxidative damage to essential components of the cell. Oxidative stress occurs when there is an imbalance in the ROS-antioxidant axis, resulting in damage to the cell that eventually can culminate in cell death.

Oxidative stress is prevalent in an array of diseases, including obesity, T2D, and AD. Much of the research into the connections between oxidative stress and insulin resistance have utilized skeletal muscle as a model tissue; however, the role of ROS in the development of brain insulin resistance has more recently gained attention. The brain is particularly sensitive to oxidative damage due to its high oxygen utilization and relatively low antioxidant activity relative to other tissues [115,134,135]. The presence of insulin resistance in the brain has also been linked to increased oxidative stress and altered antioxidant expression and activity as well as elevated levels of protein, lipid, and DNA oxidation products (Figure 3) [115,[136], [137], [138], [139]]. Treating cultured neurons with PA to mimic aspects of HFD feeding induces both oxidative stress and NF-κB signaling [115,119,140,141]. Ceramide exposure in neurons also leads to oxidative stress, insulin resistance, and mitochondrial dysfunction [115,142,143]. Further, disrupting mitochondrial homeostasis in the hypothalamus through knockdown of the mitochondrial chaperone Hsp60 is sufficient to induce insulin resistance, mitochondrial dysfunction, and ROS production [144].

Feeding mice a HFD, which increases whole-body insulin resistance (as measured by the homeostatic model of insulin resistance, HOMA-IR), positively correlates with brain ROS production, but negatively correlates with brain mitochondrial ATP production [108,138]. Ruegsegger et al. [108] found decreased activity of the antioxidant enzymes SOD2 and CAT in whole-brain lysates of mice fed a HFD for 4 weeks, consistent with some previous reports [145], but in disagreement with others [138]. However, the latter study placed mice on a HFD for 8 weeks and also showed regional differences in antioxidant enzyme expression and activity [138]. Together, these findings demonstrate the importance of considering the length of the HFD and the brain regions assessed when making conclusions regarding oxidative stress and HFD-induced brain insulin resistance. Nonetheless, the positive correlation between HOMA-IR in mice and ROS production in the brain is notable. Altogether, it is clear that oxidative stress is an important factor in brain insulin resistance that can influence and be influenced by the lipid content and inflammatory and metabolic states of the brain during insulin-resistant conditions.

### 4.2 Alzheimer's disease and insulin resistance

Insulin resistance in the brain has been observed in association with HFD feeding, obesity, and T2D [108,146,147]. Interestingly, it has also been associated with several neurologic disorders, including depression, Parkinson's disease, cognitive decline, and AD [148]. In AD, brain insulin resistance is a prominent characteristic and potential factor in the onset, independent of coincident T2D [111,149]. Studies assessing brains from recently deceased AD patients and age-matched controls found that IR and IGF1R expression were significantly reduced in multiple regions of the AD brain [150] and components of the insulin signaling cascade decreased in both T2D and AD [151]. Moreover, ex vivo stimulation of AD brains with insulin or IGF1 revealed that signaling is impaired for both hormones in AD [111]. However, T2D and excess caloric intake are also risk factors for developing cognitive deficits and AD [[152], [153], [154]], and the exact molecular mechanisms linking insulin resistance to AD are not currently well characterized. It remains unclear whether insulin resistance seen in the brain in AD occurs by the same mechanisms as T2D and HFD feeding. Wakabayashi et al. [155] attempted to address this question by comparing HFD feeding to IRS2 KO in an AD mouse model that induced amyloid-β (Aβ) accumulation. They found that AD mice fed a HFD were insulin resistant prior to the onset of amyloid pathology and that HFD feeding exacerbated the rate of Aβ accumulation [155], consistent with previous studies [156,157]. They also found that KO of IRS2, while inducing insulin resistance in the brain, liver, and pancreas, diminished Aβ deposition in the brain, suggesting that HFD feeding and IRS2 KO act through different mechanisms to impact AD pathology [155].

Although the disease's underlying pathogenesis remains unclear, the core pathological features of AD are extracellular plaques composed of Aβ and the accumulation of phosphorylated tau into intracellular tangles. Insulin signaling appears to be important for mitigating amyloid plaques in animal and cellular models of AD and also regulates normal clearance of Aβ oligomers. Whole-brain IRKO mice (NIRKO) have increased levels of phosphorylated tau, which is a critical component for the generation and accumulation of neurofibrillary tau tangles [94]. Supplementation with either insulin or IGF1 decreased cognitive deficits in an AD mouse model and increased trafficking and clearance of Aβ [[158], [159], [160], [161]]. Furthermore, insulin can ameliorate Aβ oligomer-induced impairment to long-term potentiation in hippocampal brain slices [162]. Studies conducted on primary neurons found that treating cells with Aβ oligomers induced local insulin resistance by reducing the amount of IR on dendrites prior to dendritic spine loss and sequestered the IRs to the neuronal soma [163,164]. Treating these cells with insulin stopped the loss of synapses induced by Aβ oligomer exposure, although it should be noted that the insulin concentration used in this study was high enough to activate both IR and IGF1R, possibly suggesting that IGF1R signaling is involved in preventing neuronal synapse loss in response to Aβ oligomers [4,163,165].

How and when insulin resistance arises during the course of cognitive decline and AD pathology is not currently well known; however, Aβ, ceramides, ROS, and inflammation have been suggested as mechanisms that can further the progression of AD [139,157,158,[166], [167], [168]]. It is likely that the development of insulin resistance in AD is multifactorial and highly complex.

### 4.3 Impact of brain insulin resistance on brain metabolism

As diseases associated with insulin resistance are primarily metabolic disorders, it is not surprising that metabolism and mitochondrial function in the brain are impaired in these conditions. HFD-induced brain insulin resistance leads to decreased OXPHOS and TCA cycle function in the hypothalamus, hippocampus, and cortex of mice, which coincides with reduced mitochondrial content and mRNA expression of OXPHOS components [108]. In addition, insulin-resistant rodents have increased ROS production and impaired mitochondrial oxygen consumption in the brain, resulting in reduced ATP production and mitochondrial dyshomeostasis [115,[169], [170], [171]]. These effects are also observed in AD models, where excess ROS and disruptions to both mitochondrial function and quality control are prominent aspects of the disease [172,173].

Another contributing factor to the TCA cycle and overall mitochondrial bioenergetic function is the oxidation of fatty acids. Fatty acid β oxidation is a metabolic pathway that utilizes fatty acids to generate acetyl-CoA, which is then fed into the TCA cycle. In the brain, this process is necessary to minimize the damaging accumulation of lipids. Evidence for this comes from models aimed at disrupting hypothalamic fatty acid sensing and oxidation. Knockout of carnitine palmitoyltransferase 1c (CPT1c), a brain-specific isoform of the enzyme, is necessary for transport of long-chain fatty acyl-CoAs into the mitochondria for FAO and decreases food intake and body weight [174]. This suggests that CPT1c activity in the brain modulates feeding behaviors and possibly body fat accumulation. Supporting this, increasing the amount of malonyl-CoA, a fatty acid synthesis intermediate and inhibitor of CPT1c and FAO, also reduces food intake [175]. Interestingly, astrocytes appear to utilize FAO as an energy source much more than neurons [53], which may in part be due to their position at the BBB and resulting exposure to circulating fatty acids. When FAO is overwhelmed by chronic HFD intake, increased PA in the brain impairs astrocyte lipid sensing and uptake [176]. This deficiency in astrocyte lipid uptake can lead to hypothalamic insulin resistance and accumulation of ceramides [116,176,177]. While the pathways are still not fully elucidated, cellular and mitochondrial metabolism in the brain are clearly impacted by insulin resistance.

Regarding mitochondrial quality control, some studies showed that mitochondrial fission in the hippocampus increased in response to HFD feeding [108]. Indeed, using an inhibitor of Drp1 to decrease mitochondrial fission prevents HFD-induced insulin resistance in the dorsal vagal complex of mice [145], a brain region involved in regulating hepatic glucose production [178]. Furthermore, the inhibition of Drp1 activity in primary hippocampal neurons isolated from obese (*ob/ob*) mice ameliorated the obesity-related decrease in ATP production [179], suggesting that Drp1-mediated fission may underlie deficits in neuronal ATP production in this model. Pharmacological inhibition of Drp1 in these obese (*ob/ob*) mice restored hippocampal synaptic plasticity, linking excess mitochondrial fission to obesity-related cognitive deficits [179]. However, there is likely a heterogeneous mitochondrial response to HFD feeding, as AgRP neurons have elevated mitochondrial fusion after long-term HFD exposure, which coincides with a higher AgRP firing rate [101,180,181] (Figure 2). When mitochondrial fusion is impaired in these neurons by AgRP-specific MFN1 or MFN2 KO, the mice are protected from the effects of HFD feeding. These mice demonstrate increased mitochondrial fission in AgRP neurons, leading to diminished ATP production, action potential firing, food intake, and fat mass, but increasing whole-body insulin sensitivity [101]. Disrupting mitochondrial fusion in POMC neurons impairs their ability to regulate whole-body metabolism [102,103]. Combining all these data, it is clear that there are likely cell type-specific responses to insulin resistance and nutrient availability as well as region-specific responses. As such, studies assessing overall levels of mitochondrial fission or fusion in whole-brain regions during insulin resistance may be missing important information regarding these responses’ cell-type specificity. Furthermore, how changes in mitochondrial dynamics due to HFD feeding and insulin resistance differ across brain regions requires additional research.

Altogether, the metabolic impact of insulin resistance and impaired insulin signaling in the brain is extensive. Abnormalities to mitochondrial bioenergetics and dynamics have been observed in models of insulin resistance and AD in vitro and in vivo. The contribution to these defects and whether metabolism in distinct cell types is differentially affected by insulin resistance and insulin-resistant diseases requires further exploration. Furthermore, identifying how insulin modulates metabolic processes in the brain and individual cell types and how these are affected by insulin resistance may yield novel targets for therapeutic intervention.

## 5 The therapeutic potential of diabetes treatments for neurologic disorders

### 5.1 Insulin

With the knowledge that the brain is insulin resistant in Alzheimer's disease and that improving insulin signaling in the brain in AD mouse models ameliorates the disease, a variety of approaches has been utilized to overcome the insulin signaling defects of AD. These interventions lean heavily on our knowledge of treatments for type 2 diabetes. Trials to date can be thought of broadly as attempts to either overcome insulin resistance with additional insulin, improve insulin sensitivity, or a combination of the two.

While it is known that impaired insulin signaling occurs in the brain parenchyma as a result of excess FFAs, inflammation, and ROS production, insulin transport across the BBB may also be diminished during insulin resistance. Both IR downregulation and altered IR signaling by brain endothelial cells could reduce insulin uptake into the brain; however, studies have shown that BBB integrity during insulin-resistant conditions is actually decreased, allowing the passage of more solutes into the brain [182]. Human data examining CSF insulin levels in AD have been mixed. An early study found that CSF insulin was reduced in moderate to severe AD, despite increased blood insulin levels [183], but a more recent study found no correlation between disease state and CSF insulin [184]. Further, in the second study, a higher CSF insulin concentration was associated with worse cognition and increased phosphorylated tau in women. This was in contrast to a series of studies in which inducing hyperinsulinemia with hyperinsulinemic-euglycemic clamps actually improved cognition in patients with AD [185]. However, the clamp studies rather than being an observed correlation were a potential intervention.

In T2D management, high doses of systemic insulin are often required to overcome insulin resistance and normalize blood glucose levels. The same approach cannot be used in AD patients without diabetes to overcome brain insulin resistance, as systemic treatment with insulin would result in hypoglycemia. To circumvent this issue, AD intervention trials were designed that delivered insulin directly to the brain through intranasal administration. Initial studies showed that the delivery of intranasal insulin had the potential to improve both cognition and AD biomarkers [[186], [187], [188], [189]]. These data were in line with studies that also demonstrated enhancements in memory in cognitively normal individuals receiving intranasal insulin [190]. Unfortunately, a recent phase 2/3 clinical trial designed to prove the efficacy of intranasal insulin for treating AD failed to show a meaningful difference between treatment groups [191]. The authors cited issues with the delivery device as a concern in the study, but there were a variety of other factors that could have contributed to the outcome. There may be significant differences in the degree of brain insulin resistance in a given individual, as is observed in the periphery. If that is the case, then individualized dosing could be required, but we currently have no way to determine how insulin resistant a given person's brain might be. While this might lead one to conclude that higher doses would be better, increased insulin binding to the IR leads to receptor downregulation, potentially worsening resistance [192]. Further complicating dosing of insulin in the brain, there is actually a greater abundance of IGF1R in the brain compared to IR. Both insulin and IGF1 can bind to each other's receptors and, like the IR, the IGF1R is also downregulated in AD [111]. Increased IGF1R signaling has also been shown to improve AD in mouse models [159]. If the IGF1R plays an import part in AD pathology, then even higher doses of insulin would be required due to its lower affinity for the IGF1R. Paradoxically, decreasing IGF1R signaling has also been beneficial in mouse models of AD, demonstrating a need for further research and understanding of the role that IGF1R signaling plays in AD pathology [193].

### 5.2 Insulin sensitizers

Another approach that has been attempted in recent years is improving brain IR sensitivity. Large studies have been conducted with the biguanide metformin and the thiazolidinedione (TZD) pioglitazone. Epidemiological data relating metformin to the prevention of AD have been mixed, although generally favoring a protective effect [[194], [195], [196], [197]]. This drug's mechanism of action is not entirely understood; however, it seems to improve insulin sensitivity in the liver and AMP-activated protein kinase (AMPK) signaling [198]. It also has the often overlooked side effect of causing vitamin B12 deficiency in some patients [199]. Given the relationship between vitamin B12 deficiency and cognitive impairment, this may be a reason for some of the confounding results with metformin for the prevention of AD. Several prospective intervention trials are being planned or are underway to systematically assess metformin's potential to impact aspects of aging, including cognitive dysfunction.

Pioglitazone, in contrast to metformin, has a well-defined mechanism of action. It is a peroxisome proliferator-activated receptor γ (PPARγ receptor agonist). Activation of the receptor leads to lipogenesis, removing FFAs from circulation and thus improving insulin sensitivity. This medication is a very potent insulin sensitizer and does not cause hypoglycemia in people without diabetes, making it an attractive candidate for preventing AD. However, a large prospective clinical trial to evaluate pioglitazone for preventing mild cognitive impairment was terminated early for lack of efficacy (NCT01931566). It is perhaps not all that surprising that pioglitazone was unsuccessful at preventing cognitive decline in a nondiabetic patient population. While brain insulin resistance in AD has been observed, it seems likely that Aβ and local inflammation are also important drivers of brain insulin resistance that are unlikely to be impacted by pioglitazone. While pioglitazone may be able to reduce the added burden of AD seen in people with obesity and diabetes, who are more likely to have a contribution to insulin resistance from dyslipidemia, this has not been directly tested.

More recently, there has been interest in using a newer class of diabetes drugs, sodium glucose transporter 2 (SGLT2) inhibitors, to prevent and/or treat Alzheimer's disease. This drug class reduces blood glucose by inducing glucosuria; hence, the mechanism of action is independent of insulin. However, the impact of glucose lowering may serve to reduce brain insulin resistance [200,201]. Epidemiologic evidence supports a potential benefit from this drug class on cognitive decline in patients with diabetes [202], but prospective clinical trials have not been performed. Interestingly, these drugs also cause an increase in circulating levels of ketone bodies, a preferred energy source for the brain. There are currently two small clinical trials underway to test the impact of this drug class on brain function, one with a focus on ketone body production in normal subjects (NCT03852901) and the other testing cognitive function in patients diagnosed with Alzheimer's disease (NCT03801642).

### 5.3 Combination therapy

The two most successful interventions to slow AD to date that addressed brain insulin resistance might be considered combination therapies in which insulin sensitivity and insulin secretion were likely both improved through the intervention. These included lifestyle interventions and the glucagon-like peptide-1 (GLP1) receptor agonist dulaglutide. The landmark FINGER study (Finnish Geriatric Intervention Study to Prevent Cognitive Impairment and Disability) demonstrated that targeting diet and exercise, but also cognitive and social activities and vascular risk factors, could significantly prevent cognitive decline in a high-risk population [203]. The FINGER study was not designed to isolate the effects of any of the individual interventions. While brain insulin signaling may have improved using this multifactorial approach, other risk factors were also mitigated, likely contributing to the positive result.

GLP1 receptor agonists are examples of drugs that improve insulin secretion by binding to the GLP1 receptor on pancreatic beta cells and stimulating insulin release while also decreasing insulin resistance, albeit indirectly, by binding GLP1 receptors in the hypothalamus to induce weight loss. In addition to these drugs’ potential beneficial impact on insulin signaling, GLP1 receptors are expressed throughout the brain and are able to directly impact cellular metabolism [204]. Dulaglutide, one drug in the GLP1 receptor agonist class, was found in a clinical trial to reduce the risk of developing AD [205]. While this drug and/or drug class may positively impact brain metabolism and the risk of AD, there are many caveats worth noting. First, this was an exploratory analysis of a trial designed to assess cardiovascular outcomes, not cognitive outcomes. In addition, in contrast to the previously mentioned studies, this trial only enrolled patients with diabetes. Patients in the treatment group had better diabetes control, lost weight, and had lower blood pressure and fewer adverse cardiovascular events throughout the 5 years of follow up [206]. Thus, it is difficult to separate the potential impact on brain metabolism from improvements in vascular risk. As we consider the successes and failures in addressing the contribution of brain insulin resistance to the pathogenesis and treatment of AD, it seems clear that there is still much research needed to understand the underlying causes of abnormal brain insulin receptor signaling in this disorder.

## 6 Conclusion

In the century since insulin was introduced for clinical care, our knowledge of how this hormone works has grown immensely. Despite all this progress, our understanding of insulin's action in the brain still lags well behind the rest of the body. As we continue to further delineate the role of insulin signaling in the brain, we hope that this will eventually lead us toward new therapies for obesity, diabetes, and neurodegenerative diseases.

## Author contributions

All the authors contributed equally to writing this manuscript.
